# Development and Validation of a Histologic Respiratory Index (HRI) in Poultry

**DOI:** 10.3390/pathogens14080727

**Published:** 2025-07-23

**Authors:** Tamer A. Sharafeldin, Mohamed Selim, Noreen Bashir, Sunil K. Mor

**Affiliations:** Animal Disease Research & Diagnostic Laboratory, Department of Veterinary and Biomedical Sciences, South Dakota State University, Brookings, SD 57007, USA

**Keywords:** respiratory viral diseases, experimental infection, histologic respiratory index, aMPV, LPAI, scoring

## Abstract

Respiratory viral diseases infecting poultry lead to variable lesions in the respiratory organs, including nasal sinuses, trachea, lungs, and air sacs. Additional involvement of eyelids/conjunctiva was reported. The distribution and the intensity of lesions depend on multiple factors, including virulence, the host’s immunity, and secondary or concurrent infections. It may be challenging to detect remarkable lesions during experimental infections conducted in a controlled environment because some viruses fail to produce the intense lesions seen in field cases. This creates a challenge in developing a reliable model to study pathogenicity or vaccine efficacy experimentally. The development of the proposed histologic respiratory index (HRI) aims to help monitor the least microscopic changes that can be scored, thereby creating an objective and accurate grading of lesions in experimentally infected birds. HRI scores the changes in eyelids/conjunctiva and respiratory mucosa, including hyperplasia, metaplasia, inflammatory cellular infiltration in the submucosa, including lymphocytes and heterophils, and vascular changes (vasculitis) in nasal sinuses, trachea, and lungs. The score was validated in birds infected experimentally with avian metapneumovirus (aMPV) and low pathogenic avian influenza (LPAI-H4N6). The HRI reliably graded higher scores in the respiratory organs of experimentally infected birds compared with non-infected control ones. The HRI is the first of its type with poultry viral respiratory pathogens and it was initially proven to be a reliable in pathogenicity and vaccine trials of certain poultry respiratory viral diseases.

## 1. Introduction

Respiratory viral diseases in poultry have been implicated in significant mortalities and economic losses in the poultry industry worldwide for decades [1]. The financial losses are associated with concurrent or secondary infections, which result in significant clinical disease and lesions [2]. However, experimental infection to study viral pathogenesis and vaccine safety/efficacy may not produce the significant lesions seen in the field situation [3,4,5]. This challenge creates a need for an objective grading scheme to score subtle histopathological changes that may go unnoticed in controlled experimental trials. The histologic respiratory index (HRI) is designed to assess multiple histopathologic features that are expected to change after experimental viral infection in poultry.

The histopathological changes in the affected tissues provide significant information about the pathogenicity, virulence, tissue tropism, and comparative pathogenicity of various pathogens. This helps understand the dynamics, clinical severity, and mechanism of infection progression [6]. The histological lesion assessment can also serve as an objective tool, a scoring system, to evaluate the efficacy of vaccines and the development of an integrated challenge model [7].

Previous research recruited histopathological scoring in determining the pathogenicity of various respiratory pathogens in poultry, including avian metapneumovirus [8,9,10] and Infectious Bronchitis virus [11] as well as low pathogenic avian influenza [12,13]. However, using a standardized and objective scheme for most respiratory pathogens can be more helpful, easier, and efficient.

A previously developed specific scoring system for the hock joints and gastrocnemius tendons, known as histologic inflammation scores, to evaluate the extent of turkey reovirus infection enabled the understanding of disease pathogenicity [14]. This scoring system represents a unique and objective tool, not only in the field of turkey reovirus diagnosis but also in research studies. This histologic inflammation score was effectively utilized to understand the pathogenicity of turkey reoviruses, as well as any newly emerged variants and their host ranges [15,16]. It also helped to determine the dynamics of infection and associated immunity [17]. Moreover, it was a crucial tool in the challenge model, which was used to assess the developed vaccines against turkey reoviruses [18].

Therefore, this work explains the detailed HRI and the validation in birds inoculated experimentally with two poultry respiratory viruses. The validated HRI may be used in experimental pathogenicity and vaccine evaluation trials.

## 2. Materials and Methods

### 2.1. Grading Scheme

**Figure 1:**
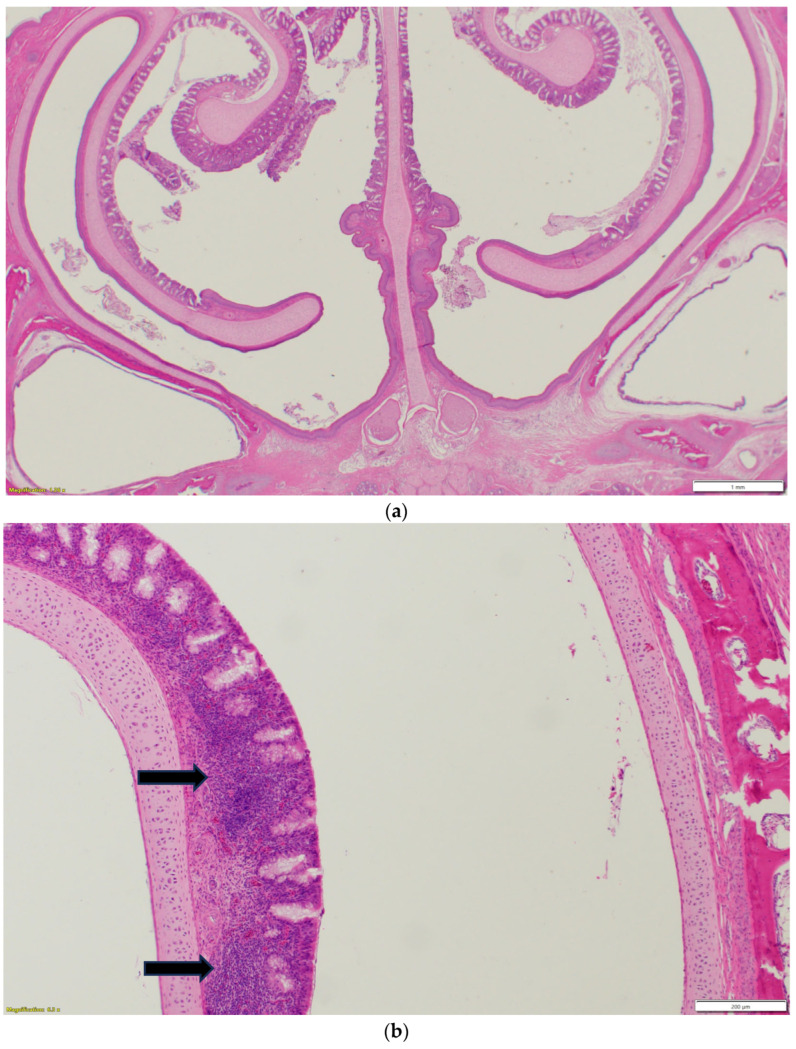

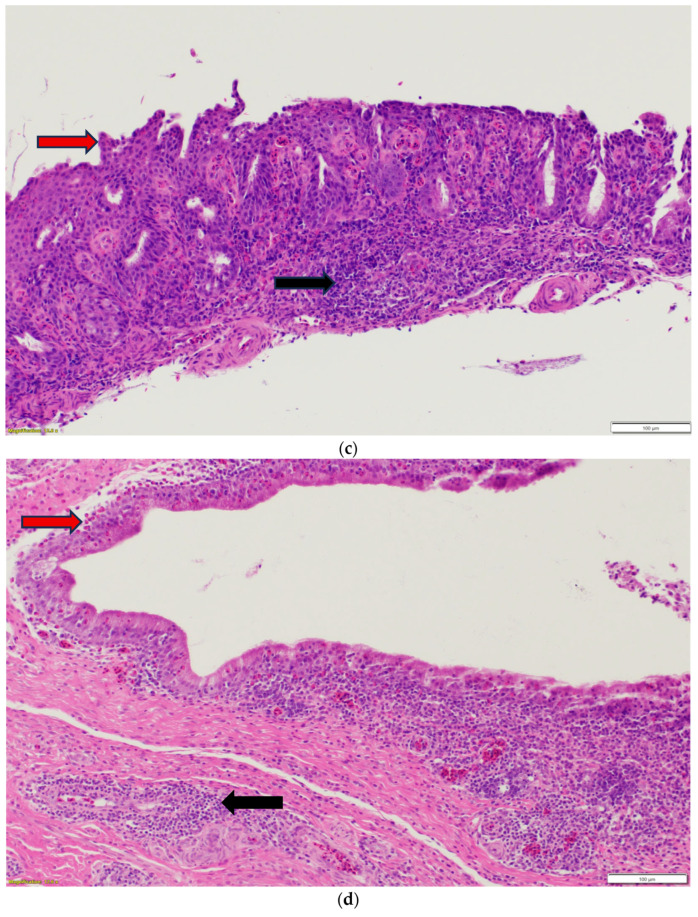

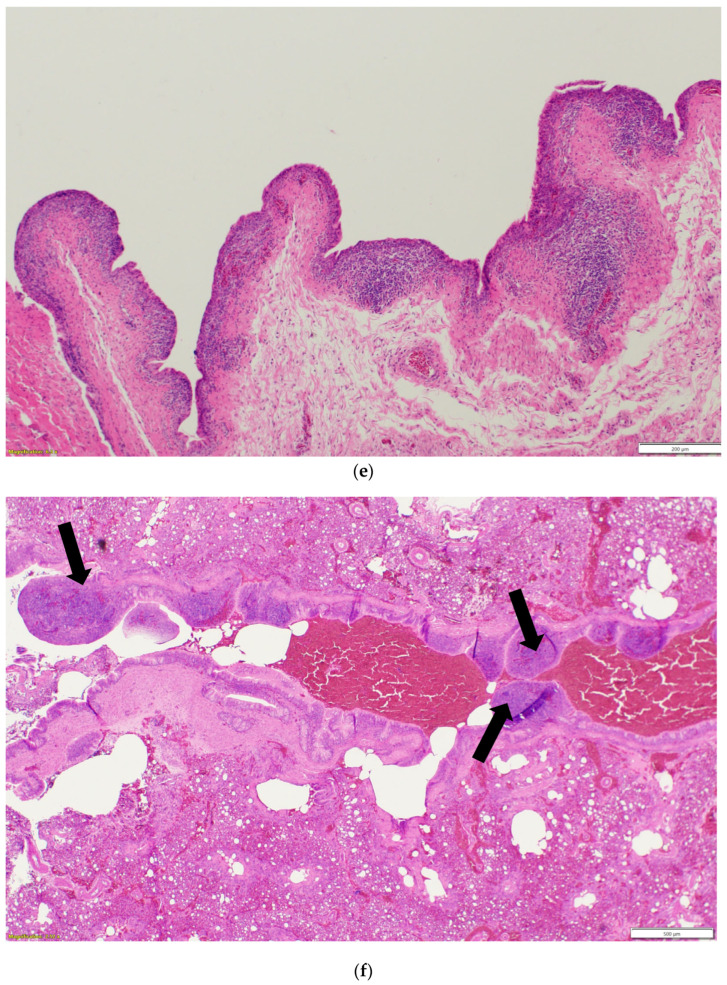

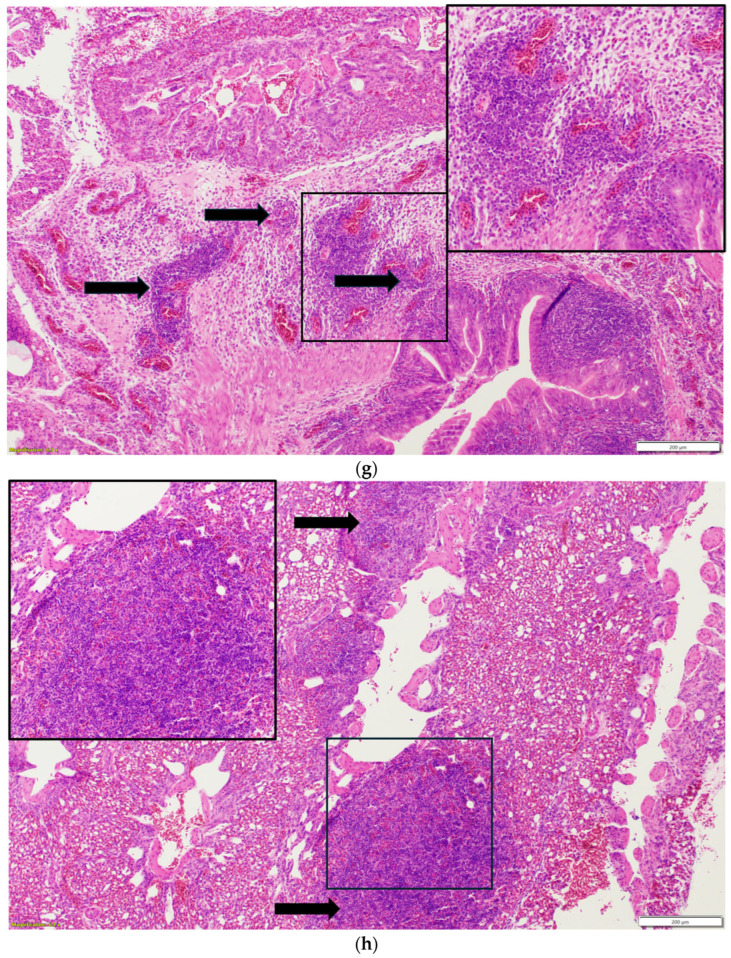
(**a**). Normal nasal and infraorbital sinuses. Ten-day-old negative control turkey. (**b**). Lymphocytic infiltration (Black arrows) in the submucosa of the nasal sinus. Seventeen-day-old aMPV-B-infected turkey at 10 days post infection. (**c**). Nasal respiratory mucosa hyperplasia and metaplasia (Red arrow), along with lymphocytic infiltration (Black arrow) in the submucosa of the nasal sinus. Seventeen-day-old turkey aMPV-infected turkey at 10 days post infection. (**d**). Heterophilic infiltration (Red arrow) in the mucosa and submucosa of the infraorbital sinus, mixed with lymphocytic infiltration as well as vasculitis (Black arrow). Fourteen-day-old LPAI-H4N6-infected turkeys at 7 days post infection. (**e**). Multifocal lymphocytic infiltration and vasculitis in the conjunctival submucosa of 14-day-old LPAI-H4N6-infected turkey at 7 days post infection. (**f**). Bronchial-associated lymphoid tissue (BALT) hyperplasia (Black arrows) around the secondary bronchi (Lungs). Fourteen-day-old LPAI-H4N6-infected turkeys at 7 days post infection. (**g**). Multifocal vasculitis (Black arrows) around the secondary bronchi (Lungs). Fourteen-day-old LPAI-H4N6-infected turkeys at 7 days post infection. (**h**). Multifocal lymphocytic infiltration (Black arrows) in the air capillaries around the parabronchus (Lungs). Fourteen-day-old LPAI-H4N6-infected turkeys at 7 days post infection.

Nasal sinuses (Figure 1a–d), including the nasal turbinate and infraorbital sinus, are scored at two levels: mucosa and submucosa. Respiratory mucosa hyperplasia/metaplasia takes 1 point, while desquamated mucosa within the lumen takes 1 more point. Submucosal lymphocytes take 1 point with a small number of lymphocytes and 2 points if they are too numerous to count (above 100 in one 400X microscopic field). An additional 1 point is added when there is infiltration of heterophils in the submucosa or mucosa. If there is vasculitis in the submucosa, 1 point is counted. The scores of the nasal turbinate and infraorbital sinuses are added to give total nasal sinus scores. Similarly, trachea and eyelids (Figure 1e) are graded considering mucosal hyperplasia/metaplasia, luminal debris, submucosal lymphocytes, heterophils, and vasculitis (1 point each). Lungs (Figure 1f–h) are scored considering hyperplasia in the bronchial associated lymphoid tissue (BALT; 1 point), germinal center formation (1 point), lymphocytic infiltration in air capillaries (1 point or 2 points in more than 100 in one 400X microscopic field), and vasculitis (1 point) (Figure 1a–h). The sum of scores of the nasal sinuses, trachea, lungs, and eyelids forms the value of the histologic respiratory index (HRI) (Table 1).

### 2.2. Validation of HRI (Experimental Design) 

Nasal sinuses, trachea, lungs, and eyelids from experimentally infected birds were scored in comparison to non-infected controls. A total of four trials were conducted where turkeys and chickens were inoculated with avian metapneumovirus (aMPV subgroup B- two trials in turkeys and one trial in chickens) and low pathogenic avian influenza (LPAI-H4N6- one trial in turkeys). One hundred (60 turkeys and 40 chickens) were inoculated in two trials (10 groups: 5 challenged and 5 control, 10 birds each) with 200 µL of 10^5^ TCID_50_ of aMPV subgroup B via oral/nasal route at different ages (1-, 7-, and 14-day-old turkeys, 7- and 14-day-old chickens) and were euthanized (3 birds/group) by CO_2_ followed by sample (Nasal sinuses, trachea, eyelids, and lungs) collection at 3, 5, and 7 days post inoculation (DPI) and fixed in formalin followed by tissue processing, paraffin embedding, sectioning on a glass slide, and stained by Hematoxylin and Eosin (H&E) for histological evaluation. In another trial, 20 turkeys (2 groups: 1 challenged and 1 control, 10 birds each) were inoculated with 200 µL of 10^5^ TCID_50_ of aMPV subgroup B at 7 days of age via the oral/nasal route, and euthanasia (10 birds/group: 1 challenged and 1 control) by CO_2_ with sample collection was performed at 10 DPI. Using another respiratory virus, turkeys were inoculated with 200 µL of 10^5^ TCID_50_ of LPAI-H4N6 via oral/nasal route at 7 days old, and birds were euthanized (10 birds/group) by CO_2_ and samples (Nasal sinuses, trachea, eyelids, and lungs) were collected and fixed in formalin followed by tissue processing, paraffin embedding, sectioning on a glass slide, and stained by Hematoxylin and Eosin (H&E)for histologic evaluation at 7 DPI. An Olympus BX43 and DP74 Olympus camera (Olympus, Center Valley, PA, USA) were used to examine and take photomicrographs. All slides were scored blindly by an American College of Veterinary Pathologists (ACVP) board certified veterinary pathologist. In all experiments, oropharyngeal/choanal swabs were taken from all birds for the detection of virus shedding.

All experiments were conducted upon the approval of the Institutional Animal Care and Use Committee (IACUC) protocol (Approval number: 2408-073A). The research team followed the guidelines of the Animal Resource Wing at South Dakota State University.

### 2.3. Statistical Analysis

Non-parametric statistics (Mann–Whitney U test) was recruited to assess the significance of difference of lesion scores between challenged and non-challenged control groups.

## 3. Results

Experimentally infected birds with aMPV and LPAI displayed a clinical disease including unilateral or bilateral nasal exudate, mouth breathing, and frothy eye secretions. No mortality was reported in challenged birds up to 7–10 days post challenge. Non-infected control did not display any of these respiratory symptoms. Both aMPV and LPAI were detected in tracheal swabs of challenged birds. There was no evidence of bacterial infection in experimental birds.

### 3.1. Scores in aMPV Infected Turkeys (Trial1)

**Figure 2:**
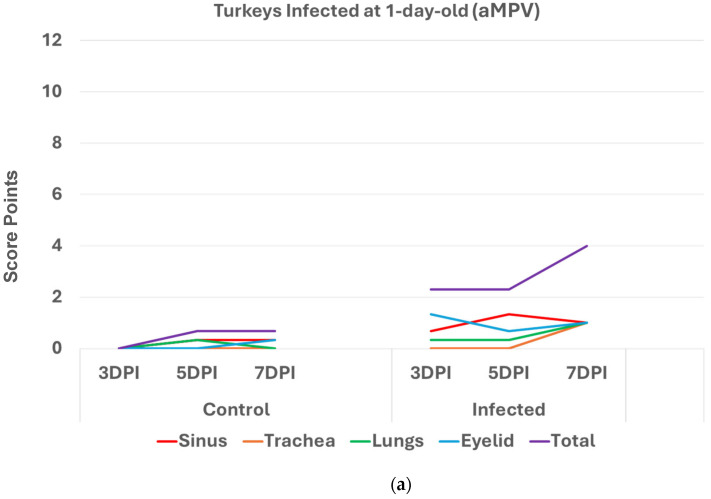

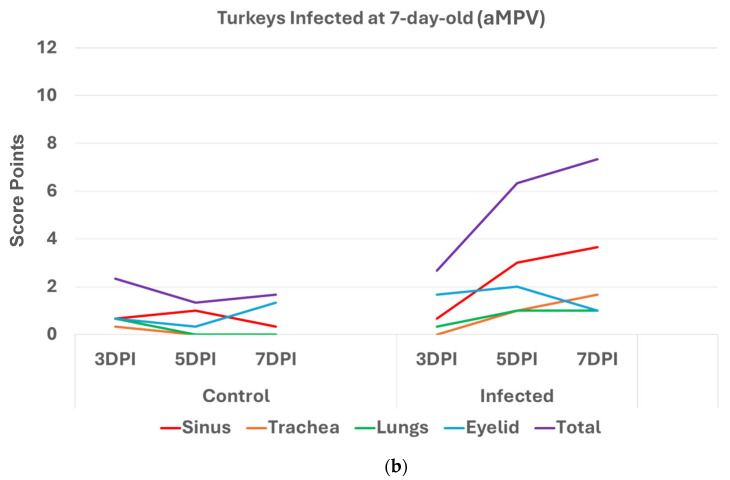

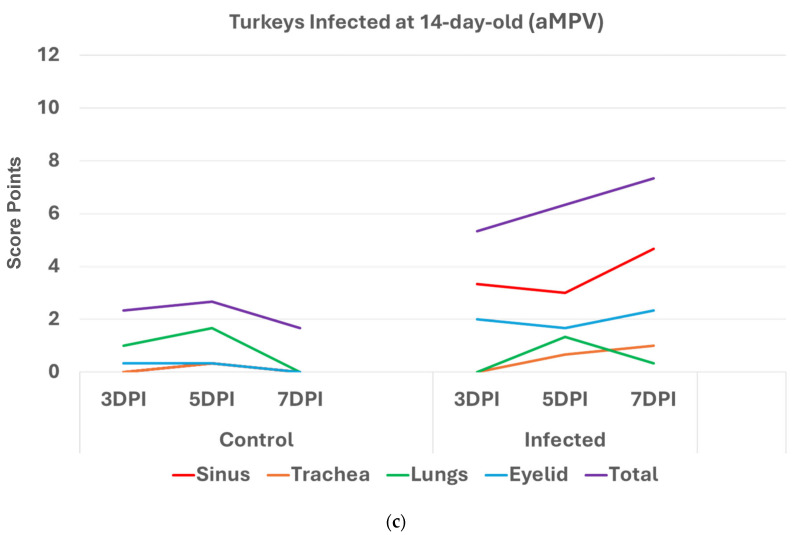
(**a**). Histologic respiratory index (HRI) at 3, 5, and 7 days post infection (DPI) (3 birds a group per timepoint) in turkeys inoculated with aMPV at 1 day old. Total HRI, sinus, and eyelid scores are statistically significantly higher (*p* < 0.05) in infected birds compared with non-infected controls at 3 DPI, 5DPI and 7DPI (Mann–Whitney U test). (**b**). Histologic respiratory index (HRI) at 3, 5, and 7 days post infection (DPI) (3 birds a group per timepoint) in turkeys inoculated with aMPV at 7 days old. Total HRI, sinus, and trachea scores are statistically significantly higher (*p* < 0.05) in infected birds compared with non-infected controls at 5DPI and 7DPI (Mann–Whitney U test). (**c**). Histologic respiratory index (HRI) at 3, 5, and 7 days post infection (DPI) (3 birds a group per timepoint) in turkeys inoculated with aMPV at 14 days of age. Total HRI, sinus, eyelid, and trachea scores are Statistically significantly higher (*p* < 0.05) in infected birds compared with non-infected controls at 3DPI, 5DPI, and 7DPI (Mann–Whitney U Test).

Turkeys infected with aMPV at 1 day old (Figure 2a) had a statistically significantly higher (*p* < 0.05 Mann–Whitney U test) total HRI compared with non-infected controls at 3, 5, and 7 days post infection (DPI). The gap between the two groups increased significantly as the birds aged. Sinus and eyelids scores were the main contributors to the difference between the control and infected groups. A similar pattern was recorded/observed in groups inoculated at 7 and 14 days old (Figure 2b,c) with a statistically significantly higher score (*p* < 0.05). Sinus was the central part of the score difference, followed by the eyelids.

### 3.2. Scores in aMPV Infected Chickens (Trial2)

**Figure 3:**
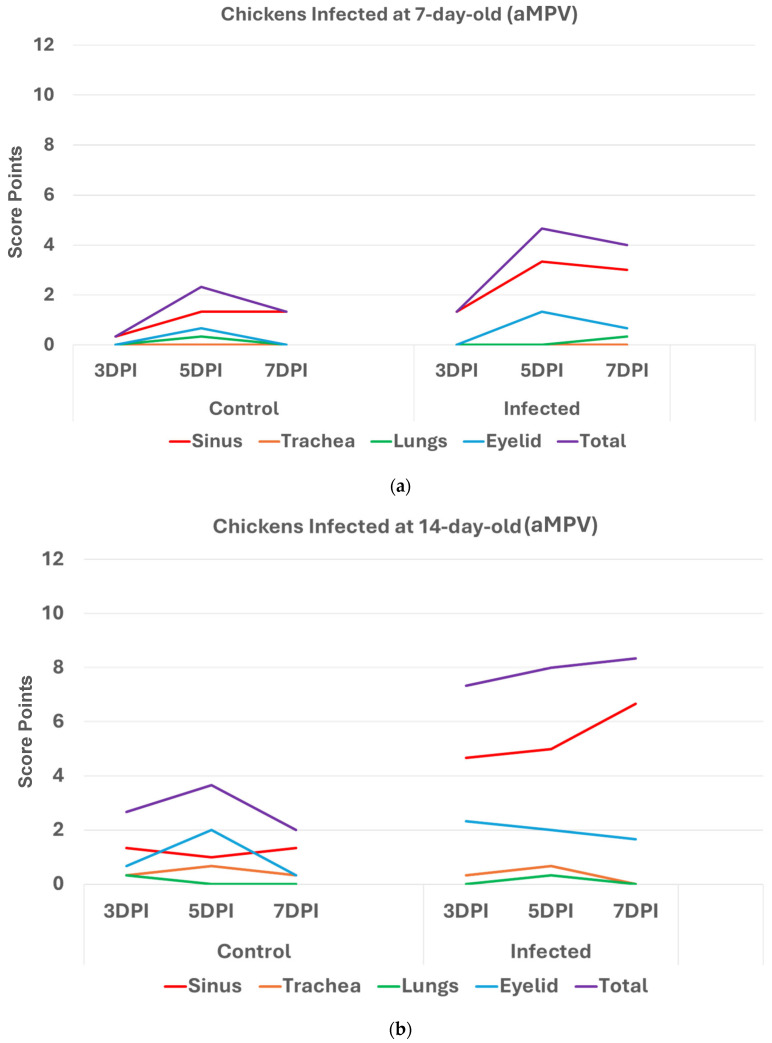
(**a**). Histologic respiratory index (HRI) at 3, 5, and 7 days post infection (DPI) (3 birds a group per timepoint) in chickens inoculated with aMPV at 7 days old. Total HRI, sinus, and eyelid scores are statistically significantly higher (*p* < 0.05) in infected birds compared with non-infected controls at 5DPI and 7DPI (Mann–Whitney U Test). (**b**)**.** Histologic respiratory index (HRI) at 3, 5, and 7 days post infection (DPI) (3 birds a group per timepoint) in chickens inoculated with aMPV at 14 days of age. Total HRI, sinus, and eyelid scores are statistically Significantly higher (*p* < 0.05) in infected birds compared with non-infected controls at 3DPI, 5DPI, and 7DPI (Mann–Whitney U Test).

Chickens infected with aMPV at 7 and 14 days old (Figure 3a,b): Statistically significantly higher (*p* < 0.05) HRI in challenged chickens compared with non-infected control chickens was observed in with aMPV challenged at 7 and 14 days old. Similarly, sinuses and eyelids created the main difference between the control and infected groups.

### 3.3. Scores in aMPV Infected Turkeys (Trial3)

**Figure 4:**
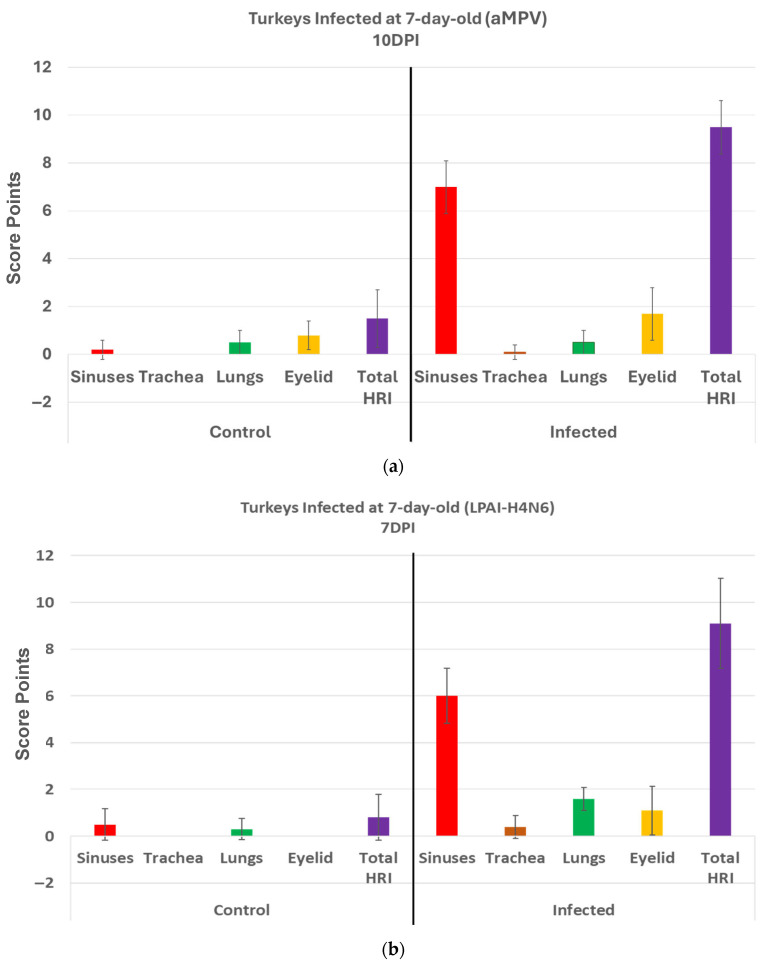
(**a**). Histologic respiratory index (HRI) at 10 days post infection (DPI) (10 birds per group) in turkeys inoculated with aMPV at 7 days of age. Total HRI, and sinuses scores are statistically significantly higher (*p* < 0.05) in infected birds compared with non-infected controls (Mann–Whitney U Test). (**b**). Histologic respiratory index (HRI) at 7 days post infection (DPI) (10 birds per group) in turkeys inoculated with low pathogenic avian influenza (LPAI-H4N6) at 7 days of age. Total HRI, sinuses, trachea, lungs, and eyelid scores are statistically significantly higher (*p* < 0.05) in infected birds compared with non-infected controls (Mann–Whitney U Test).

Turkeys inoculated with aMPV at 7 days of age showed statistically significantly higher lesion scores (HRI) (*p* < 0.05) at 10 DPI compared with the non-infected control group. Sinus and eyelids were the main organs scoring points compared with other organs (Figure 4a).

### 3.4. Scores in LPAI-H4N6 Infected Turkeys (Trial4)

Turkeys inoculated with low pathogenic avian influenza (LPAI-H4N6) at 7 days of age had also statistically significantly higher HRI at 7 DPI compared with the non-infected control group. Sinus, lungs, eyelids, and trachea were orderly contributing to a higher total HRI (Figure 4b).

## 4. Discussion

The development of an objective histologic score for respiratory organs in poultry is necessary for a detailed and unbiased evaluation of disease pathogenicity. It is also a potential tool in establishing the challenge model needed for any vaccine evaluation. While conducting respiratory viral pathogenicity trials under a controlled environment, observing striking lesions may not be achieved due to the absence of secondary bacterial infection in the experimentally infected birds [19]. In addition, experimentally infected birds may recover after a few days with no observable lesions or clinical signs of disease [20]. That is why using a validated histologic score for respiratory organs is strongly recommended to accurately monitor microscopic changes in a detailed measurable pattern. The HRI not only provides a sum of scores for different respiratory organs, considering expected cellular changes after being infected with respiratory viral pathogens in poultry, but it also offers detailed information about lesion intensity, progression, and distribution in various respiratory organs.

The score takes into account a variety of mucosal and submucosal changes that occur during and after respiratory viral infections. Respiratory mucosal cells hyperplasia, metaplasia, breakdown, and subsequent formation of luminal debris are subsequent changes seen during respiratory viral infection in poultry [13,21,22]. Scoring respiratory mucosal cellular changes is representative of viral pathogenicity, and the additional score for the luminal cellular debris after respiratory mucosal cell breakdown refers to the intensity of viral virulence [23,24]. In addition, submucosal lymphocytic infiltration (1 point or 2 points due to excessive lymphocytes) as well as an additional point for mucosal/submucosal heterophilic infiltration is a correct referral to the intensity of the host response, which is proportional to virulence or involvement in a mild secondary infection [25,26]. Adding a point for vasculitis is a fair representation of respiratory viral pathogenesis.

Respiratory viral pathogenicity is to be assessed via different features compared within the respiratory organs. In lungs, BALT hyperplasia reflects an immune response to infection or vaccination [27]. An additional point regarding germinal center formation explains both the intensity and duration of the immune response. Furthermore, lymphocytic infiltration within air capillaries and/or vasculitis in avian lungs are observed features with respiratory viral infections in poultry [28].

Analyzing the scores of different organs involved in the HRI enables an understanding of the significant parts of the HRI. This can help with the slight modification of the grading scheme to obtain the best scoring criteria for different respiratory diseases. In aMPV, relying mainly on the sinuses and eyelids, and less likely on the trachea and lungs, can support an accurate grading for evaluation of pathogenicity. Similarly, in LPAI-H4N6, relying mainly on sinuses and eyelids is helpful, but with the addition of lungs to the list, in which scores are significantly higher in infected birds compared with non-infected controls at 7DPI (Figure 4b).

The nearly similar pattern in inoculated chickens and turkeys with aMPV (Figure 2 and Figure 3) and in turkeys inoculated with aMPV and LPAI-H4N6 (Figure 4) confirms the reliability of the developed HRI. Further validation and refinement are ongoing with additional experimental trials using different respiratory pathogens, at various ages and routes of inoculation, and at other times post inoculation.

## 5. Conclusions

The developed and validated HRI can be reliably used in grading lesions in experimentally infected birds. Validation with aMPV and LPAI-H4N6 was established. Further validation and minor modifications will be needed for other poultry respiratory viral diseases. The developed HRI can also be recruited in future challenge models for vaccine trials. It can also be used with other poultry respiratory viral pathogens upon future validations.

## Figures and Tables

**Table 1:** **HRI scoring criteria**. Different lesions and scores used in the calculation of the final histologic respiratory index (HRI). TNTC (too numerous to count).

Scoring Criteria		Lymphocytes		Heterophils	Vasculitis	Hyperplasia/ Metaplasia	Luminal Debris
		A Few	TNTC				
Nasal Sinuses	Turbinate	1	2	1	1	1	1
	Infraorbital sinus	1	2	1	1	1	1
Trachea		1	2	1	1	1	1
Eyelids/Conjunctiva		1	2	1	1	1	1
Lungs		Air capillary Lymphocytes		BALT Hyperplasia 1	1	Germinal Center 1	
		1	2				

## Data Availability

The original contributions presented in this study are included in the article. Further inquiries can be directed to the corresponding author.

## References

[1] Liu H., Pan S., Wang C., Yang W., Wei X., He Y., Xu T., Shi K., Si H. (2025). Review of Respiratory Syndromes in Poultry: Pathogens, Prevention, and Control Measures. Vet. Res..

[2] Samy A., Naguib M.M. (2018). Avian Respiratory Coinfection and Impact on Avian Influenza Pathogenicity in Domestic Poultry: Field and Experimental Findings. Vet. Sci..

[3] Kariithi H.M., Welch C.N., Ferreira H.L., Pusch E.A., Ateya L.O., Binepal Y.S., Apopo A.A., Dulu T.D., Afonso C.L., Suarez D.L. (2020). Genetic Characterization and Pathogenesis of the First H9N2 Low Pathogenic Avian Influenza Viruses Isolated from Chickens in Kenyan Live Bird Markets. Infect. Genet. Evol..

[4] Kye S.J., Park M.J., Kim N.Y., Lee Y.N., Heo G.B., Baek Y.K., Shin J.I., Lee M.H., Lee Y.J. (2021). Pathogenicity of H9N2 Low Pathogenic Avian Influenza Viruses of Different Lineages Isolated from Live Bird Markets Tested in Three Animal Models: SPF Chickens, Korean Native Chickens, and Ducks. Poult. Sci..

[5] Brown P.A., Allée C., Courtillon C., Szerman N., Lemaitre E., Toquin D., Mangart J.M., Amelot M., Eterradossi N. (2019). Host Specificity of Avian Metapneumoviruses. Avian Pathol..

[6] MacLachlan N.J., Dubovi E.J. (2017). Pathogenesis of Viral Infections and Diseases. Fenner’s Veterinary Virology.

[7] Gibson-Corley K.N., Olivier A.K., Meyerholz D.K. (2013). Principles for Valid Histopathologic Scoring in Research. Vet. Pathol..

[8] Aung Y.H., Liman M., Neumann U., Rautenschlein S. (2008). Reproducibility of Swollen Sinuses in Broilers by Experimental Infection with Avian Metapneumovirus Subtypes A and B of Turkey Origin and Their Comparative Pathogenesis. Avian Pathol..

[9] Youn H.-N., Noh J.-Y., Kim M.-S., Ju H.-S., Park D.-H., Lee D.-Y., Kim K.-J., Go S.-H., Song C.-S. (2020). Efficacy of a Novel Avian Metapneumovirus Live Vaccine Candidate Based on Vaccination Route and Age. Poult. Sci..

[10] Meng L., Yu M., Wang S., Chen Y., Bao Y., Liu P., Feng X., He T., Guo R., Zhang T. (2024). A Novel Live Attenuated Vaccine Candidate Protects Chickens against Subtype B Avian Metapneumovirus. J. Integr. Agric..

[11] Bande F., Arshad S.S., Omar A.R., Bejo M.H., Abubakar M.S., Abba Y. (2016). Pathogenesis and Diagnostic Approaches of Avian Infectious Bronchitis. Adv. Virol..

[12] Begum J.A., Hossain I., Nooruzzaman M., King J., Chowdhury E.H., Harder T.C., Parvin R. (2023). Experimental Pathogenicity of H9N2 Avian Influenza Viruses Harboring a Tri-Basic Hemagglutinin Cleavage Site in Sonali and Broiler Chickens. Viruses.

[13] Bóna M., Földi J., Dénes L., Harnos A., Paszerbovics B., Mándoki M. (2023). Evaluation of the Virulence of Low Pathogenic H9N2 Avian Influenza Virus Strains in Broiler Chickens. Vet. Sci..

[14] Sharafeldin T.A., Mor S.K., Bekele A.Z., Verma H., Goyal S.M., Porter R.E. (2014). The Role of Avian Reoviruses in Turkey Tenosynovitis/Arthritis. Avian Pathol..

[15] Sharafeldin T.A., Mor S.K., Verma H., Bekele A.Z., Ismagilova L., Goyal S.M., Porter R.E. (2015). Pathogenicity of Newly Emergent Turkey Arthritis Reoviruses in Chickens. Poult. Sci..

[16] Kumar R., Sharafeldin T.A., Sobhy N.M., Goyal S.M., Porter R.E., Mor S.K. (2022). Comparative Pathogenesis of Turkey Reoviruses. Avian Pathol..

[17] Sharafeldin T.A., Mor S.K., Sobhy N.M., Xing Z., Reed K.M., Goyal S.M., Porter R.E. (2015). A Newly Emergent Turkey Arthritis Reovirus Shows Dominant Enteric Tropism and Induces Significantly Elevated Innate Antiviral and T Helper-1 Cytokine Responses. PLoS ONE.

[18] Kumar R., Porter R.E., Mor S.K., Goyal S.M. (2022). Efficacy and Immunogenicity of Recombinant Pichinde Virus-Vectored Turkey Arthritis Reovirus Subunit Vaccine. Vaccines.

[19] Trottein F., Alcorn J.F. (2019). Editorial: Secondary Respiratory Infections in the Context of Acute and Chronic Pulmonary Diseases. Front. Immunol..

[20] Spickler A.R., Trampel D.W., Roth J.A. (2008). The Onset of Virus Shedding and Clinical Signs in Chickens Infected with High-Pathogenicity and Low-Pathogenicity Avian Influenza Viruses. Avian Pathol..

[21] Histopathological Profiling of Respiratory Tract Lesions in Chickens. https://www.researchgate.net/publication/281004753_Histopathological_Profiling_of_Respiratory_Tract_Lesions_in_Chickens.

[22] Benyeda Z., Szeredi L., Mató T., Süveges T., Balka G., Abonyi-Tóth Z., Rusvai M., Palya V. (2010). Comparative Histopathology and Immunohistochemistry of QX-like, Massachusetts and 793/B Serotypes of Infectious Bronchitis Virus Infection in Chickens. J. Comp. Pathol..

[23] Okino C.H., Mores M.A.Z., Trevisol I.M., Coldebella A., Montassier H.J., Brentano L. (2017). Early Immune Responses and Development of Pathogenesis of Avian Infectious Bronchitis Viruses with Different Virulence Profiles. PLoS ONE.

[24] Rüger N., Sid H., Meens J., Szostak M.P., Baumgärtner W., Bexter F., Rautenschlein S. (2021). New Insights into the Host–Pathogen Interaction of Mycoplasma Gallisepticum and Avian Metapneumovirus in Tracheal Organ Cultures of Chicken. Microorganisms.

[25] Maina J.N. (2023). A Critical Assessment of the Cellular Defences of the Avian Respiratory System: Are Birds in General and Poultry in Particular Relatively More Susceptible to Pulmonary Infections/Afflictions?. Biol. Rev..

[26] Campbell E.L., Kao D.J., Colgan S.P. (2016). Neutrophils and the Inflammatory Tissue Microenvironment in the Mucosa. Immunol. Rev..

[27] Pabst R. (2022). The Bronchus-Associated-Lymphoid Tissue (BALT) an Unique Lymphoid Organ in Man and Animals. Ann. Anat.-Anat. Anz..

[28] Bezuidenhout A., Mondal S.P., Buckles E.L. (2011). Histopathological and Immunohistochemical Study of Air Sac Lesions Induced by Two Strains of Infectious Bronchitis Virus. J. Comp. Pathol..

